# Alterations in the gut microbiota of toddlers with cow milk protein allergy treated with a partially hydrolyzed formula containing synbiotics: A nonrandomized controlled interventional study

**DOI:** 10.1002/fsn3.3801

**Published:** 2023-11-14

**Authors:** Mengyao Qian, Wei Liu, Xueying Feng, Zhaochuan Yang, Xiaomei Liu, Liang Ma, Yanchun Shan, Ni Ran, Mingji Yi, Changlong Wei, Chenyang Lu, Yanxia Wang

**Affiliations:** ^1^ The Affiliated Hospital of Qingdao University Qingdao China; ^2^ Shanghai Genecobio Company Ltd. Shanghai China; ^3^ School of Marine Science Ningbo University Ningbo China

**Keywords:** cow milk protein allergy, gut microbiota, partially hydrolyzed formulas, prebiotic, probiotic

## Abstract

Formulas containing intact cow milk protein are appropriate alternatives when human milk (HM) is not feasible. However, for babies with a physician‐diagnosed cow milk protein allergy (CMPA), hydrolyzed formulas are needed. We conducted a 3‐month, open‐label, nonrandomized concurrent controlled trial (ChiCTR2100046909) between June 2021 and October 2022 in Qingdao City, China. In this study, CMPA toddlers were fed with a partially hydrolyzed formula containing synbiotics (pHF, *n* = 43) and compared with healthy toddlers fed a regular intact protein formula (IF, *n* = 45) or HM (*n* = 21). The primary endpoint was weight gain; the secondary endpoints were changes in body length and head circumference of both CMPA and healthy toddlers after 3‐month feeding; and the exploratory outcomes were changes in gut microbiota composition. After 3 months, there were no significant group differences for length‐for‐age, weight‐for‐age, or head circumference‐for‐age *Z* scores. In the gut microbiota, pHF feeding increased its richness and diversity, similar to those of IF‐fed and HM‐fed healthy toddlers. Compared with healthy toddlers, the toddlers with CMPA showed an increased abundance of phylum Bacteroidota, Firmicutes, class Clostridia, and Bacteroidia, and a decreased abundance of class Negativicutes, while pHF feeding partly eliminated these original differences. Moreover, pHF feeding increased the abundance of short‐chain fatty acid producers. Our data suggested that this pHF partly simulated the beneficial effects of HM and shifted the gut microbiota of toddlers with CMPA toward that of healthy individuals. In conclusion, this synbiotic‐containing pHF might be an appropriate alternative for toddlers with CMPA.

## Graphical abstract

A 3‐month, open‐label, nonrandomized concurrent controlled trial in Qingdao City, China, was conducted to compare the growth and gut microbiota composition between CMPA toddlers fed a partially hydrolyzed formula containing synbiotics and healthy toddlers fed a regular intact protein formula or HM.

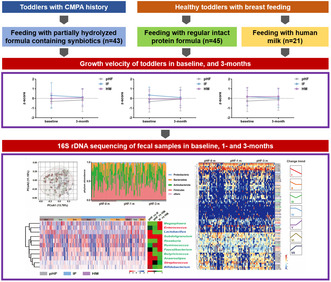

## 1 INTRODUCTION

In the last 100 years, the prevalence of food allergies has increased worldwide, and some estimates suggest that up to 10% of some populations are affected (Gupta et al., 2018, 2019; Lopes & Sicherer, 2020). Food allergies begin in infancy and show adverse effects on growth and development, while severe food allergies may lead to life‐threatening complications (Seth et al., 2020). Cow milk protein allergy (CMPA) is one of the most common food allergies, especially during childhood, affecting approximately 3%–8% of the total pediatric population in different countries (Lajnaf et al., 2023). More seriously, CMPA causes the greatest number of fatal reactions in children (Abrams & Sicherer, 2021). CMPA is caused by the protein components in cow's milk, and the most common allergens for patients with CMPA are caseins (especially αs1‐casein), β‐lactoglobulin, and α‐lactalbumin (D'Auria et al., 2018). Among them, caseins are considered the main allergen due to their high content and strong heat‐stable properties (Wang et al., 2022).

Economic developments in recent decades have changed eating habits and lifestyles, leading to direct modulation of the gut microbiota (de Vos et al., 2022). Children with CMPA showed dysbiosis in the gut microbiota and its metabolites (Yu et al., 2023). Previous studies identified gut microbiota dysbiosis in infants preceding the development of food allergies (Azad et al., 2015; Savage et al., 2018), and the potential causal relationship was further revealed in the mouse model that fecal microbiota transplantation from healthy donors protected germ‐free mice against the development of CMPA (Feehley et al., 2019). Moreover, successful applications of live and heat‐killed probiotics in the management of CMPA had been observed in clinical trials (Berni Canani et al., 2012; Strisciuglio et al., 2023; Yamamoto‐Hanada et al., 2023), which indicated both probiotics and their derived metabolites and cellular components might contribute to CMPA alleviation. In addition, short‐chain fatty acids (SCFAs) are some of the most abundant gut microbiota metabolites in the colon. Different fecal SCFA levels were observed between healthy and CMPA infants (Thompson‐Chagoyan et al., 2011), which were reported to alleviate food allergy by repairing epithelial barrier function, restoring the balance of T helper cells, and improving gut microbiota dysbiosis (Chen et al., 2023). Therefore, gut microbiota should be regarded as a new perspective in CMPA research.

Although the debate on whether breastfeeding is beneficial or may, to the contrary, lead to the development of CMPA has been controversial over decades, human milk (HM) is considered to be the gold standard for baby nutrition in early life. There are many studies that show the benefits of HM to protect against the development of atopic disease in infants, especially for those with atopic heredity (Burris et al., 2021). On the one hand, the allergens in HM that come from the maternal diet and are inhaled by the mother are in much smaller amounts than the antigens from cow's milk (Gamirova et al., 2022). On the other hand, the endogenous intact proteins in HM and the biologically active peptides derived from the mother's diet showed important effects from a nutritional and immunological point of view (Zhu & Dingess, 2019). Moreover, as a source of commensal bacteria (Moossavi et al., 2019), HM showed beneficial effects on the gut colonization of the baby after birth by producing antimicrobial compounds, preventing pathogenic bacterial adhesion, and stimulating intestinal mucin expression (Lyons et al., 2020), and establishing the gut microbiota in the first 1000 days had confirmed to play an irreplaceable role in health (Marchesi et al., 2016). The origin of bacteria in HM was proposed to be contamination from the baby's skin and oral cavity or translocation by the enteromammary pathway (Kordy et al., 2020). Human milk oligosaccharides (HMOs) in HM promote the growth of beneficial bacteria and inhibit the adherence of pathogens to the intestinal epithelium (Carr et al., 2021). Therefore, beneficial gut microbiota modulation is one of the key features that make HM the optimum feeding regime for babies.

However, when HM is not available, formulas are the appropriate alternatives. Continuous efforts have been made to mimic the formulas more closely for HM. In general, intact protein from cow's milk is the most widely used protein source in formulas. For babies with a CMPA, hydrolyzed cow's milk formulas, plant‐based (soy, rice, almond, etc.,), and other mammalian milk (goat, sheep, camel, etc.,) might be valuable alternatives. Among them, hydrolyzed cow's milk formulas could be further classified into three major classes, including partially hydrolyzed cow's milk protein‐based formulas (pHFs), extensively hydrolyzed cow's milk protein‐based formulas, and amino acid‐based formulas, while pHFs showed relatively better palatability than others (Verduci et al., 2019). Previous studies have reported the beneficial effects of synbiotics‐containing pHF on gastrointestinal tolerance and gut microbiota modulation in healthy infants (Kok et al., 2020; Wang et al., 2021). However, the effect of this specific pHF on the gut microbiota in toddlers with a CMPA needed to be explored.

Gut microbiota played an essential role in the pathogenesis and susceptibility of CMPA, and beneficial modulation effects on gut microbiota were regarded as an excellent property of HM. Therefore, we carried out a 3‐month, open‐label, nonrandomized controlled interventional study in Qingdao City to evaluate the alterations in the gut microbiota of a specific pHF containing synbiotics [short‐chain galactooligosaccharides (scGOS), long‐chain fructooligosaccharides (lcFOS), and *Bifidobacterium breve* M‐16V (*B. breve* M‐16V)] for toddlers with CMPA and compared them with healthy toddlers fed with HM or regular intact protein formula (IF). In this study, we hypothesized that this specific synbiotics‐containing pHF partly simulated the beneficial effects of HM on the gut microbiota and altered the gut microbiota of toddlers with CMPA toward that of healthy individuals.

## 2 METHODS

### 2.1 Institutional Review Board statement

All procedures were approved by the Institutional Review Board (IRB) of the Affiliated Hospital of Qingdao University (Approval No. of ethics committee: QYFYKLL) and are registered at the Chinese Clinical Trial Registry (www.chictr.org.cn), and the registration number is ChiCTR2100046909.

### 2.2 Study design and participants

A 3‐month, open‐label, nonrandomized, concurrently controlled trial of two formula groups and a reference HM feeding group was carried out between June 2021 and October 2022 in the affiliated hospital of Qingdao University, Qingdao City, Shandong Province, China. This study was conducted in compliance with the Declaration of Helsinki. Before enrollment, written informed consent was obtained from the parent(s)/legally accepted representative >18 years of age for each toddler.

Fifty‐three toddlers with physician‐diagnosed CMPA were enrolled. Healthy toddlers were enrolled who were within the same age range and showed no suspicion of CMPA, and all healthy toddlers received breastfeeding before enrollment. In addition, the major inclusion criteria were (1) 37–42 weeks of gestation; (2) range from 12 to 24 months at enrollment; (3) weight, length, and head circumference at enrollment within the normal range (±2 SD) according to the WHO Child Growth Standards; and (4) no current or previous illness/condition.

### 2.3 Interventions

The parents of toddlers with physician‐diagnosed CMPA who decided to exclusively consume a partially hydrolyzed cow's milk protein‐based formula with synbiotics SYNEO® (0.8 g/100 mL scGOS and lcFOS, and *B. breve* M‐16V) (Table S1) for 3 months were assigned to the pHF group (*n* = 43). Healthy toddlers' parents who decided to change from breastfeeding to exclusive consumption of a regular intact protein formula for 3 months were assigned to the IF group (*n* = 45) and those who decided to continue exclusive HM feeding for 3 months were assigned to the HM group (*n* = 21). It was suggested that parents feed the formulas to their toddlers as they deem appropriate.

### 2.4 Study objectives and endpoints

The primary objective of this study was to assess whether toddlers with CMPA who were fed with pHF had similar growth to healthy toddlers fed with IF or HM. The primary endpoint was weight gain from enrollment to 3 months of feeding. The secondary objectives included the assessment of body length and head circumference after 3 months of feeding with the WHO growth reference. In addition, the study aimed to assess the changes in the composition of the gut microbiome at baseline, 1‐, and 3‐month time points.

### 2.5 Schedule of study visits

Toddlers were fed with the pHF, IF, or HM from enrollment (baseline) to 3 months. Demographic data, growth parameters, and serum IgE information were recorded at baseline, and the growth parameters were monitored at 3‐month time points.

### 2.6 Fecal genomic DNA extraction and 16S rDNA sequencing

For all groups, fecal samples were collected at baseline and after corresponding feedings for 1 and 3 months. 16S rDNA sequencing was performed at Shanghai Majorbio Biopharm Technology Co., Ltd. (Shanghai, China) using the Illumina MiSeq platform (paired‐end sequencing, 2 × 300 bp) as previously described (Zhao et al., 2022). The genomic DNA was extracted by a QIAamp‐DNA Stool Mini Kit (Shanghai Qiagen Bioengineering Co., Ltd., Shanghai, China), and the V3‐V4 hypervariable region of 16S rDNA was amplified with primers 338F and 806R.

### 2.7 16S rDNA sequencing data analysis

Raw FASTQ files were multiplexed and filtered using QIIME (version 1.8.0). Operational taxonomic units (OTUs) were clustered by Usearch (version 7.1) at an identity level of 97%. The sequence that was most abundant in each OTU was used as the representative sequence and annotated by the Ribosomal Database Project (RDP) classifier (version 2.2) for taxonomic classification. The microbial richness and diversity indices were determined by Mothur (version 1.30.1). The phylogenetic tree and the normalized relative OTU abundance were used for weighted UniFrac principal coordinate analysis (PCoA) via the vegan package in R software (version 3.2).

### 2.8 Statistical analysis

Baseline characteristics were compared using the Fisher's exact test. The differences in α and β diversity were calculated by the nonparametric Kruskal–Wallis test with Dunn's post hoc test in GraphPad Prism (version 8.0.2; GraphPad Software Inc., San Diego, CA, USA). For single taxa at each time point, differences were assessed by one‐way analysis of variance (ANOVA) with nonparametric Wilcoxon tests for comparisons of two groups and the nonparametric Kruskal–Wallis test for comparisons of more than two groups. Spearman's correlation analysis of abundance change trends between two groups was analyzed by SPSS (version 19.0, Chicago, IL, USA). Mengyao Qian analyzed the data throughout the entire trial.

## 3 RESULTS

### 3.1 Study population

**FIGURE 1:**
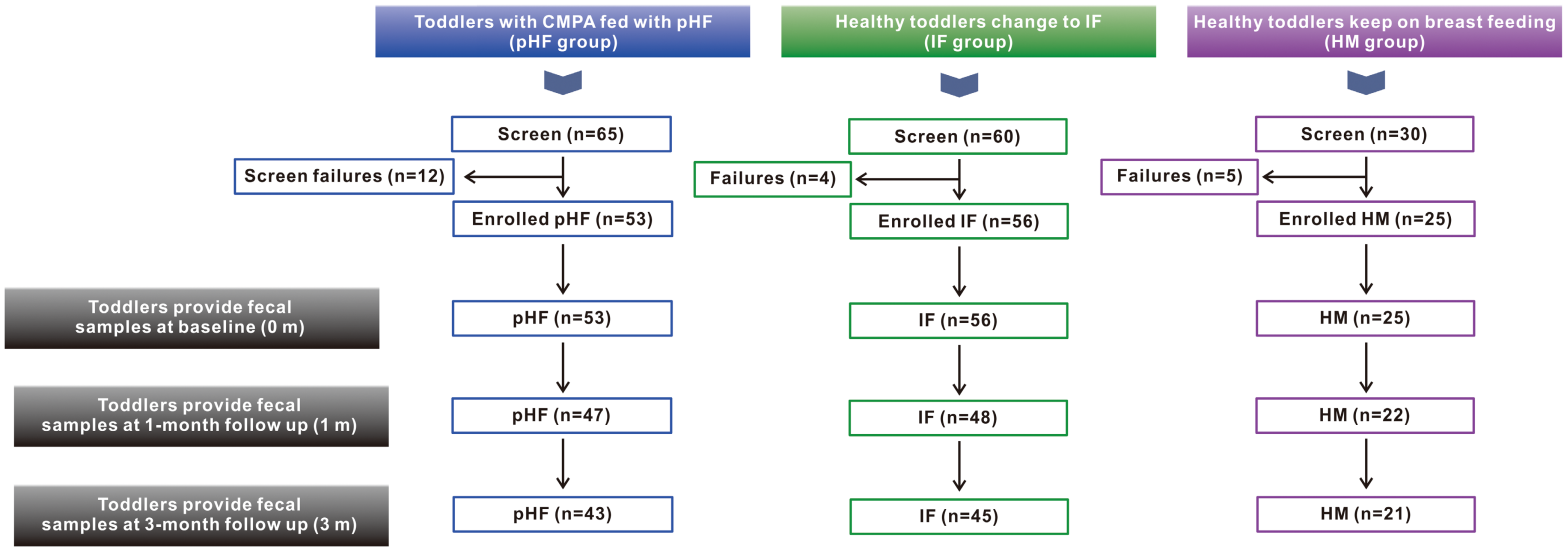
The flow chart of participants through the trial.

From June 2021 to October 2022, we enrolled 53 Chinese toddlers with physician‐diagnosed CMPA and agreed to use a partially hydrolyzed cow's milk protein‐based formula containing synbiotics (pHF group). In addition, we enrolled 81 healthy Chinese toddlers who received breastfeeding, 56 of whom switched voluntarily to regular intact cow's milk protein‐based formulas (IF group), while the remaining 25 toddlers continued breastfeeding (HM group). During the course of the study, fecal samples were collected after 1 and 3 months of feeding, and 18.8% (10 of 53), 19.6% (11 of 56), and 16% (4 of 25) of toddlers failed to follow up after 3 months of feeding in the pHF, IF, and HM groups, respectively (Figure 1).

### 3.2 Baseline characteristics

There were no significant differences in most of the characteristics (*p* > .05), while there were only significantly lower ratios of parents who had completed college in the pHF group. In addition, the toddlers in the pHF group showed higher than 0.7 IU/mL (level 2) serum IgE levels, which were considered milk allergies (Tang et al., 2022), while the serum IgE levels of toddlers in the other two groups were lower than 0.35 IU/mL (Table 1).

**TABLE 1:** Baseline characteristics of the participating toddlers.

Characteristics	pHF	IF	HM
No.	43	45	21
Infant sex, male [*n* (%)]	28 (65.1)	22 (48.9)	12 (57.1)
Maternal age at pregnancy (years)	29.86 ± 3.60	30.53 ± 3.93	29.90 ± 3.33
Gestational age (week)	39.40 ± 1.42	39.36 ± 2.00	39.29 ± 1.23
Type of delivery, vaginal [*n* (%)]	24 (55.8)	26 (57.8)	13 (61.9)
Age when recruited (month)	15.07 ± 2.58	17.07 ± 2.43	15.57 ± 3.19
Weight when recruited, kg	10.06 ± 1.34	10.95 ± 2.87	10.71 ± 1.15
Length when recruited, cm	79.31 ± 4.17	81.91 ± 3.19	80.29 ± 3.81
Head circumference when recruited, cm	46.30 ± 1.67	46.98 ± 1.36	46.99 ± 1.49
Mother education, completed college [*n* (%)]**	34 (79.1)	45 (100)	19 (90.5)
Father education, completed college [*n* (%)]*	32 (74.4)	41 (91.1)	20 (95.2)
Living environment, in town [*n* (%)]	38 (88.4)	41 (91.1)	21 (100)
Number of household members, <4 [*n* (%)]	8 (18.6)	10 (22.2)	8 (38.1)
Nation, Han [*n* (%)]	42 (97.7)	43 (95.6)	21 (100)
Serum IgE (IU/mL)***	0.943 ± 0.897	0.179 ± 0.086	0.16 ± 0.089

*p < .5, **p < .01, ***p < .001.

### 3.3 Anthropometric data

**FIGURE 2:**
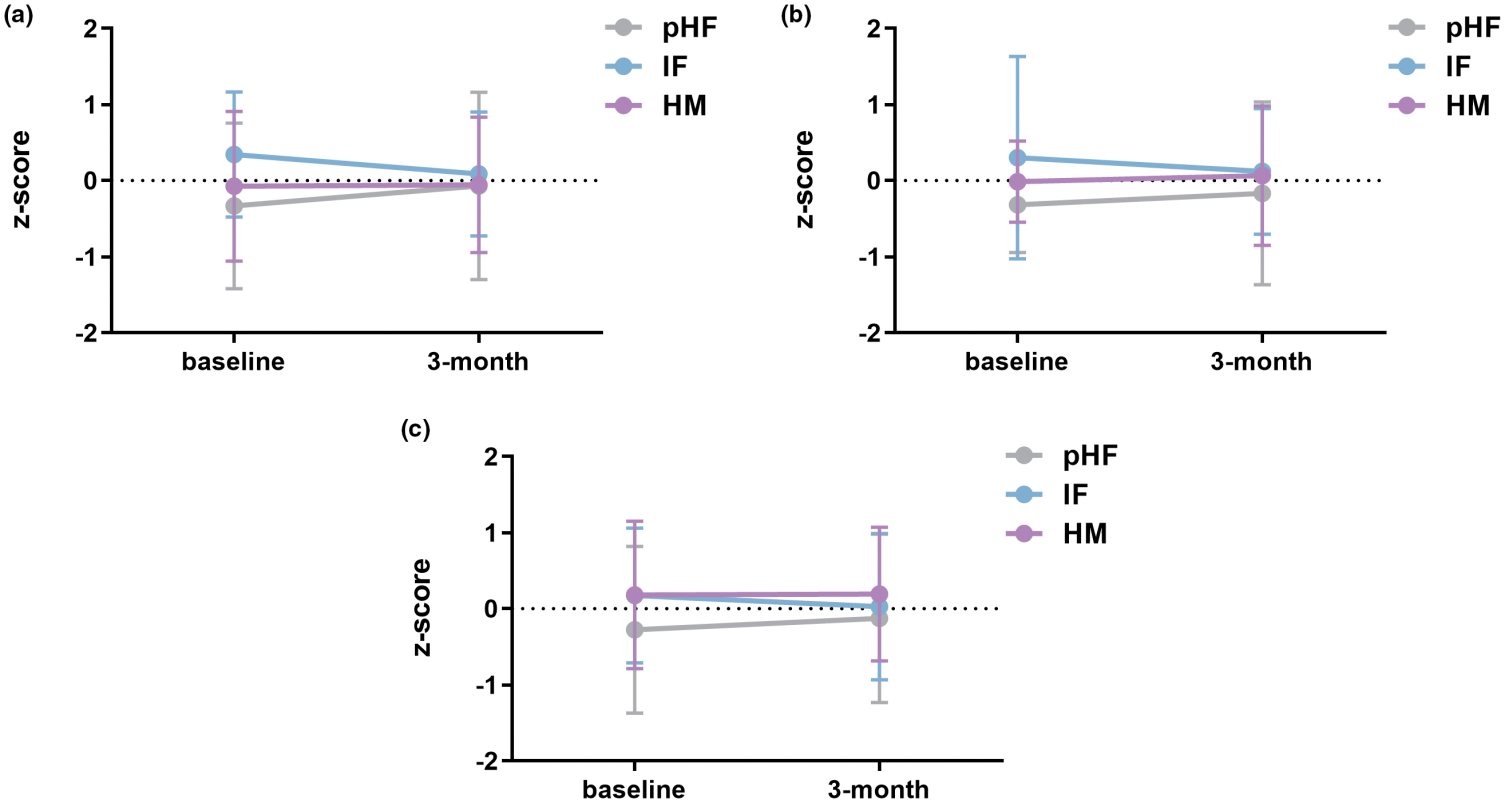
Mean *Z* scores from baseline to 3 months of age. (a) Length‐for‐age *Z* score; (b) Weight‐for‐age *Z* score; (c) Head circumference‐for‐age *Z* score.

After 3‐month feeding, the mean weight gain, linear growth, and head growth were 1.15 kg, 5.5 cm, and 1.1 cm in the pHF group, respectively, while the corresponding data was 0.31 kg, 3.6 cm, and 0.6 cm in the IF group, and 0.9 kg, 4.6 cm, and 0.9 cm in the HM group. Comparing anthropometric parameters among these three groups, no significant differences in body length, body weight, or head circumference were observed at baseline and after 3‐month feeding. In addition, there were no significant group differences for length‐for‐age, weight‐for‐age, or head circumference‐for‐age *Z* scores. In the pHF and HM groups, we observed an upward trend of all *Z* scores from baseline to 3 months, suggesting a minor increase in the growth velocity of toddlers in these two groups. However, in the IF group, we observed a downward trend (Figure 2).

### 3.4 pHF altered the gut microbiota composition of toddlers with CMPA

**FIGURE 3:**
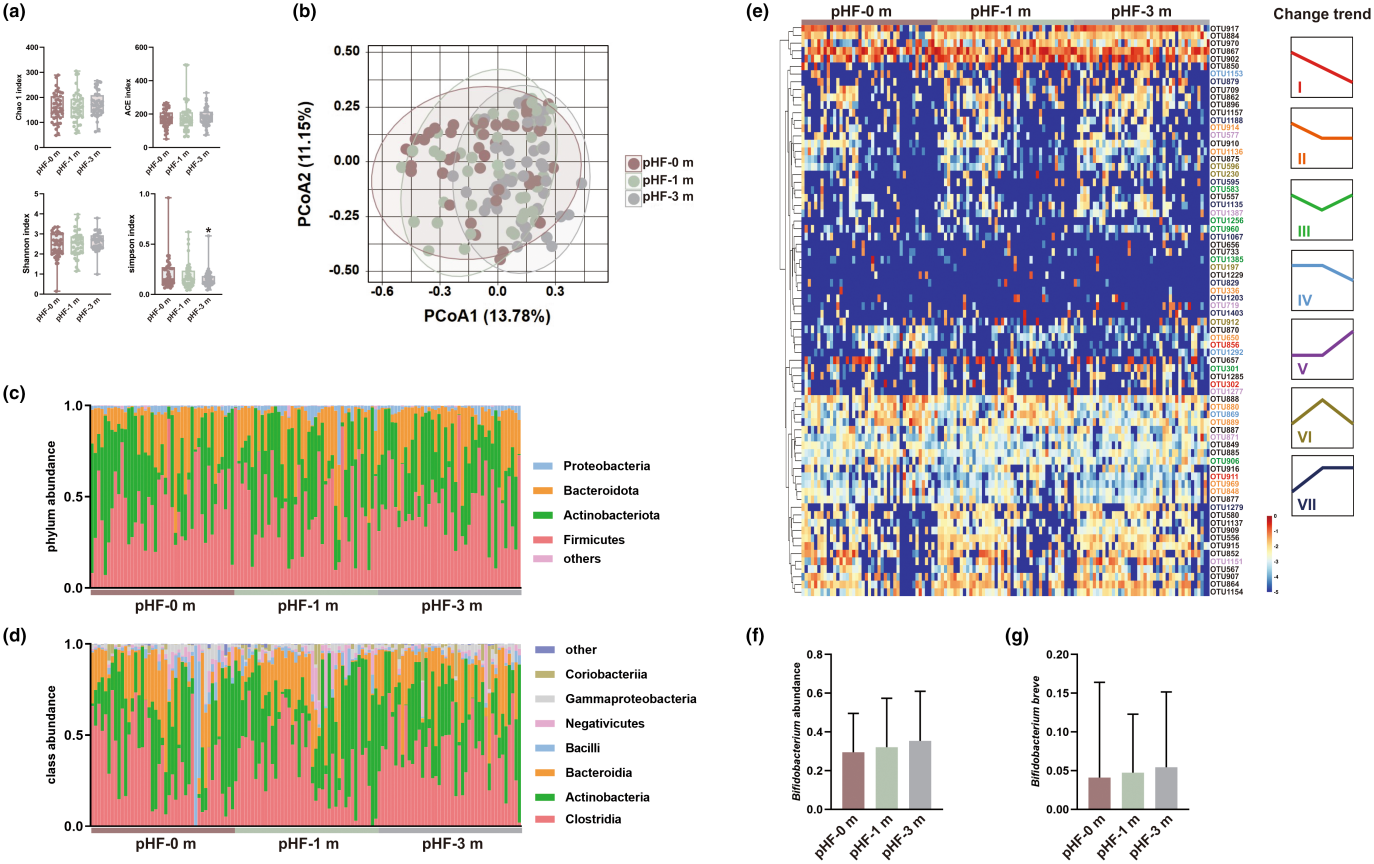
Gut microbiota composition of the toddlers with physician‐diagnosed CMPA after feeding with pHF (*n* = 43). (a) Microbial α diversity; *p < .05, compared with pHF‐0m group; (b) Microbial β diversity. Bacterial taxonomic profiling at the phylum (c) and class (d) levels; (e) 74 identified key OTUs with high abundances, which were grouped into seven clusters according to their change trends. The different colors of OTUs represent their change trends. The abundance of genus *Bifidobacterium* (f) and species *Bifidobacterium breve* (g) at three time points in the pHF group; data are presented as mean ± SD.

As shown in Figure 3a, the richness indices of the gut microbiota in allergic toddlers, including ACE and Chao1, increased over time, but the differences were not significant. In addition, the Shannon index increased while the Simpson index decreased over time, and the Simpson index reached significance after 3 months of pHF feeding compared with baseline (*p* < .05). Moreover, β‐diversity indicated that pHF feeding changed the overall structure of the gut microbiota to a certain degree, indicating that pHF feeding improved gut dysbiosis in allergic toddlers, at least in part (Figure 3b).

At the phylum level, most of the microbiota belonged to Firmicutes, Actinobacteria, and Bacteroides. Compared with the baseline, pHF feeding increased the abundance of Firmicutes and Bacteroides and decreased the Actinobacteria abundance, whereas the highest abundance of Proteobacteria was observed after 1 month of feeding (Figure 3c and Table S2). At the class level, Clostridia, Actinobacteria, and Bacteroidia accounted for more than 85% of the total abundance. Among them, pHF feeding increased the abundance of Clostridia and Coriobacteria but decreased that of Bacilli. In addition, the highest abundance of Actinobacteria and Negativicutes and the lowest abundance of Bacteroides and Gammaproteobacteria were observed in the pHF‐1m group (Figure 3d and Table S3).

Seventy‐four key OTUs with high abundance were identified in this study, and 39 of them were grouped into seven clusters by STEM analysis according to their change trends among the three groups. OTU302, OTU856, and OTU911 were grouped into Cluster 1, which decreased over time and belonged to the genera *Bacteroides*, *Clostridium* sensu stricto 1, and *Clostridium innocuum* group. Eight and three OTUs were grouped into Cluster 2 and Cluster 4, which showed partly decreasing trends among the three groups, and most of them belonged to the families Lachnospiraceae (*n* = 3) and Veillonellaceae (*n* = 2). In addition, six and nine OTUs showed partly increasing trends among the three groups and were grouped into Cluster 5 and Cluster 7, belonging to the families Bacteroidaceae (*n* = 3), Ruminococcaceae (*n* = 3), and Lachnospiraceae (*n* = 2) (Figure 3e and Table S4).

Moreover, the abundances of *Bifidobacterium* and *Bifidobacterium breve* accounted for approximately 30% and 5% of the total abundance on average (Figure 3f,g), which were increased in allergic toddlers fed pHF over time (*p* > .05).

### 3.5 pHF changed the gut microbiota of toddlers with CMPA toward that of healthy toddlers fed breast milk

**FIGURE 4:**
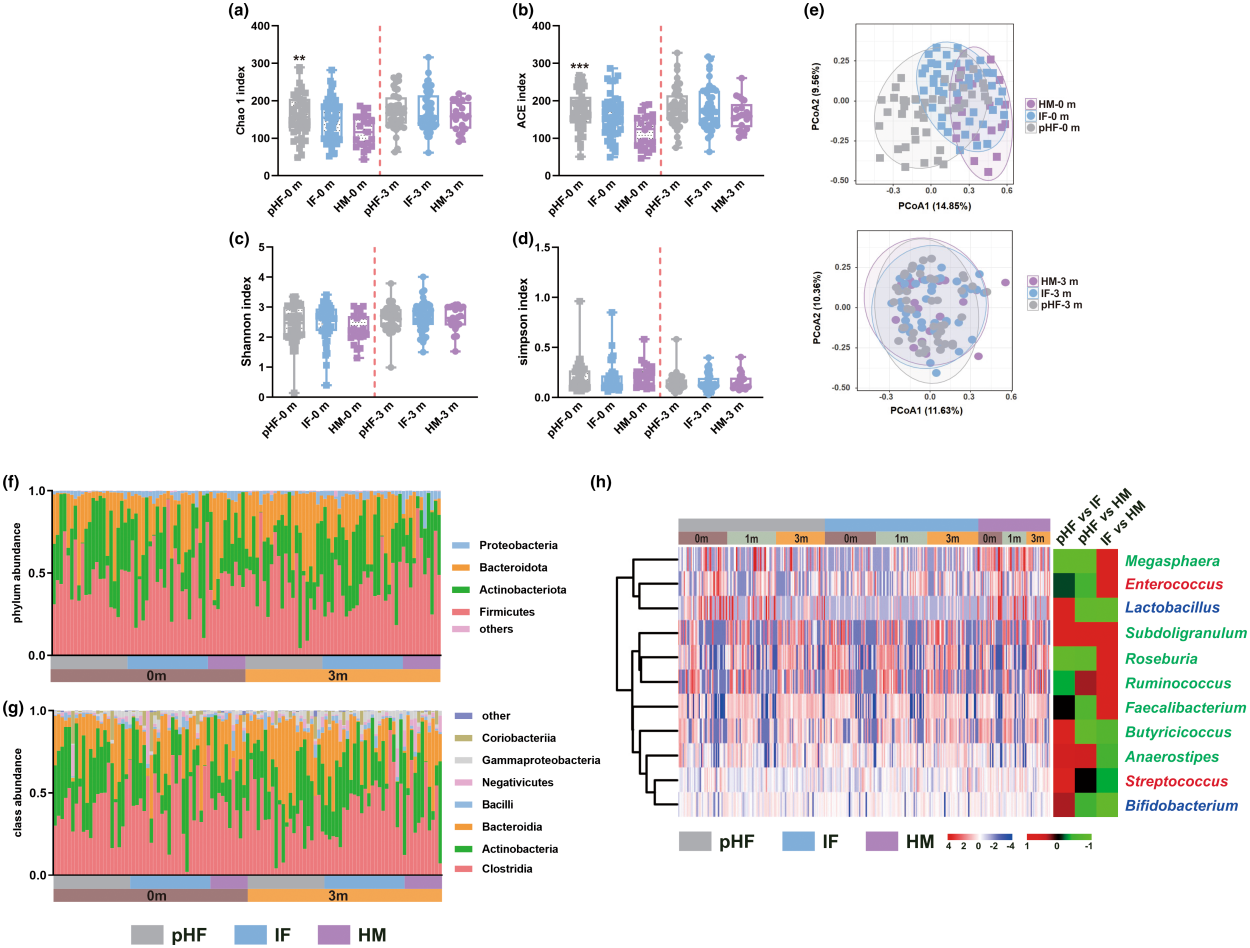
Gut microbiota composition of the allergic and healthy toddlers after feeding with pHF (*n* = 43), IF (*n* = 45) and HM (*n* = 21) for 3 months. (a–d) Microbial α diversity; *p < .05, **p < .01, ***p < .001, compared with the HM group; (e) Microbial β diversity. Bacterial taxonomic profiling at the phylum (f) and class (g) levels. (h) Distributions of probiotics and short‐chain fatty acid producers and the correlation analysis of pairwise comparisons. The middle heatmap shows the correlation coefficient value between the two groups. The genera belonging to SCFA producers and probiotics are marked with green and red, respectively, and the genera regarded as both SCFA producers and probiotics are marked with blue.

At baseline, significant differences in microbial richness were observed in the pHF group compared with the HM group (*p* < .01 and *p* < .001), whereas 3 months of feeding increased microbial richness and eliminated the differences between the pHF and HM groups (Figure 4a,b). For microbial diversity, 3 months of feeding increased the Shannon index and decreased the Simpson index but did not result in significant differences (Figure 4c,d). Moreover, the PCoA results indicated that 3 months of pHF feeding changed the gut microbiota structure to that of the HM group, indicating the beneficial effects of pHF on the gut microbiota (Figure 4e).

At the phylum level, 3 months of feeding increased the abundance of Firmicutes and decreased that of Actinobacteria. Breast milk feeding decreased the Bacteroides abundance and increased the Proteobacteria abundance, whereas completely opposite effects were observed in the IF group, and pHF feeding increased the abundance of Bacteroides and Proteobacteria. In addition, the pHF group showed relatively closer levels of Firmicutes and Bacteroides than the IF group and the HM group after 3 months of feeding (Figure 4f and Table S5). At the class level, all feedings increased the abundance of Clostridia and decreased those of Actinobacteria and Bacilli, while opposite regulatory effects were observed between formula feedings (pHF and IF groups) and breast milk feeding for Bacteroidia, Coriobacteriia, and Gammaproteobacteria. HM decreased the levels of Bacteroides and Coriobacteriia and increased the level of Gammaproteobacteria. In addition, the abundance of Negativicutes was decreased by both pHF and HM feedings and increased after IF treatment. Otherwise, the pHF group showed relatively closer levels of Clostridia, Bacteroidia, and Negativicutes than the IF group and the HM group after 3 months of feeding (Figure 4g and Table S6). In addition, 48, 38, and 45 genera with different abundances in response to different feedings between the baseline and 3‐month feeding groups were identified in this study (Figure S1a–c and Tables S7–S9), and genera *Robinsoniella*, *Hafnia Obesumbacterium*, and *Epulopiscium* were identified by all three pairwise comparisons (Figure S1d).

### 3.6 Distributions of probiotics and SCFA producers during interventions

Four probiotics and nine SCFA producers were selected to investigate their distributions in response to pHF, IF, and HM feedings. The genus *Bifidobacterium* (>25%) showed the highest abundance, followed by *Faecalibacterium* (>5%) and *Roseburia* (>1%). Among them, seven (except *Anaerostipes*, *Butyricicoccus*, *Faecalibacterium*, and *Roseburia*) showed increased abundances after 3 months of feeding with pHF compared with baseline. In addition, *Streptococcus*, *Butyricicoccus*, *Roseburia*, and *Ruminococcus* showed positively correlated change trends between pHF and HM feedings (Figure 4h and Table S10).

## 4 DISCUSSION

In this study, the gut microbiota modulation effects of specific synbiotics‐containing pHF in toddlers with physician‐diagnosed CMPA were evaluated via 16S rDNA sequencing and compared with the gut microbiota of healthy toddlers fed IF or HM. We found that this specific pHF improved the gut microbiota of toddlers with CMPA to more closely resemble that of healthy toddlers.

HM is generally believed to be preferable to other sources of milk, and formula designers aimed to mimic its composition to closely resemble that of HM. HM is a natural symbiotic containing prebiotics and probiotics. HMOs were detected only in HM, which showed high resistance to digestive enzymes and acted as a selective substrate for *Bifidobacteria* (Triantis et al., 2018). In addition, *Bifidobacterium breve*, *Lactobacillus salivarius*, and *Lactobacillus fermentum* are identified as probiotics in HM (Lyons et al., 2020). Previous studies have reported that synbiotics consisting of *B. breve* M‐16V and HMO analogs prevented the allergic response to cow's milk and whole whey protein in mice (Kostadinova et al., 2016; Sagar et al., 2014). Similar conclusions were obtained in the clinic, in which amino acid formulas containing synbiotics improved tolerance development with CMPA (Chatchatee et al., 2022). In this study, the specific pHF contained HMO analogs and *B. breve* M‐16V (Table S1), which have been proposed to have better effects than IF in improving the gut microbiota.

Due to the vital role of the gut microbiota in CMPA, fecal samples after feeding with pHF in toddlers with CMPA were sequenced. The Shannon index increased over time (Figure 3a), consistent with a result obtained from allergic infants fed an amino acid‐based formula containing synbiotics (Wopereis et al., 2019). Moreover, extensively hydrolyzed casein formula supplemented with *Lactobacillus rhamnosus* GG increased the abundances of *Blautia*, *Roseburia*, and *Coprococcus* in allergic infants. In clinical trials of allergic infants with a synbiotics‐containing amino acid‐based formula, enrichment of *Bifidobacterium* spp. and *Veillonella* sp. and inhibition of adult‐like species (*Ruminococcus* and *Alistipes*) were reported (Guest & Fuller, 2019; Wopereis et al., 2019). Similar change trends were observed in the genera *Bifidobacterium*, *Blautia*, *Veillonella*, *Roseburia*, and *Coprococcus* after pHF feeding for 3 months, whereas opposite trends were observed for *Ruminococcus* and *Alistipes*. Among them, the anti‐allergic potential of *Bifidobacterium* was widely accepted, which was mediated by modifying the skewed Th2 profile back into balance, producing antimicrobial compounds, and inhibiting the pathogenic bacteria (Shu et al., 2019). *Veillonella* metabolizes lactic acid into SCFAs and has beneficial cross‐feeding with lactate producers (Pham et al., 2017). In addition, as the tolerance development was reported to be associated with an increased level of fecal butyrate (Guest & Fuller, 2019), the abundance of SCFA producers was quantified, and butyrate producers *Bifidobacterium*, *Lactobacillus*, *Megasphaera*, *Ruminococcus*, and *Subdoligranulum* were found to be increased after 3‐month pHF feeding (Table S10).

Although various studies have aimed to investigate gut microbiota dysbiosis in allergic babies, the results were preliminary because the gut microbiota composition is profoundly influenced by multiple environmental factors. In general, a more diverse gut microbiota was reported in allergic infants (Berni Canani et al., 2016), while 3 months of pHF feeding further increased the diversity of the gut microbiota in toddlers with CMPA (Figures 3a and 4c). CMPA decreased the abundance of the phylum Bacteroidetes and increased that of Firmicutes (Akagawa & Kaneko, 2022), which was partly restored by pHF feeding for the phylum Bacteroidetes but aggravated the increase in Firmicutes (Figure 3c and Table S2). Among the previously reported genera with decreased abundances in response to food allergies, including *Citrobacter*, *Dialister*, *Dorea*, *Clostridium*, *Haemophilus*, and *Oscillospira* (Chen et al., 2016; Savage et al., 2018), 3 months of pHF feeding restored the abundances of the genera *Citrobacter*, *Dialister*, and *Dorea* to healthy levels. In addition, a reduced abundance of butyric acid‐producing bacteria, along with decreased contents of SCFAs, was a key gut microbiota feature of toddlers with CMPA. In this study, pHF feeding increased the abundances of the genera *Bifidobacterium*, *Lactobacillus*, *Megasphaera*, *Ruminococcus*, and *Subdoligranulum*; contributed to SCFA production; and showed potential antiallergic activity.

Due to the vital role of the gut microbiota in early health, the gut microbiota modulation effects of formulas also need to be considered. Although higher microbial richness and diversity were found in toddlers with CMPA than in healthy individuals, consistent with a previous report (Wopereis et al., 2019), both HM and pHF feedings increased the microbial richness and diversity (Figure 4a–d). Among the 393 genera detected in this study, 98 showed the same change trends between HM and pHF feeding. Moreover, four genera belonging to important probiotics and SCFA producers showed consistent change trends in the comparison between the HM and pHF groups (Figure 4h), indicating that pHF had similar but not identical beneficial effects on the gut microbiota compared with HM, and the microbiota profiles in response to pHF feeding showed a changing trend mimicking that of HM‐fed toddlers.

In conclusion, this study indicated that these specific pHF‐containing synbiotics offered an effective nutritional strategy to alter the gut microbiota composition in toddlers with physician‐diagnosed CMPA toward that of healthy individuals and partly simulated the beneficial effects of HM on the gut microbiota.

## AUTHOR CONTRIBUTIONS

**Mengyao Qian:** Data curation (equal); formal analysis (equal); methodology (equal); software (equal); visualization (equal); writing – original draft (equal). **Wei Liu:** Data curation (equal). **Xueying Feng:** Data curation (equal). **Zhaochuan Yang:** Formal analysis (equal). **Xiaomei Liu:** Formal analysis (equal). **Liang Ma:** Formal analysis (equal). **Yanchun Shan:** Methodology (equal). **Ni Ran:** Methodology (equal). **Mingji Yi:** Writing – review and editing (equal). **Changlong Wei:** Validation (equal). **Chenyang Lu:** Data curation (supporting); software (equal). **Yanxia Wang:** Funding acquisition (equal); project administration (equal); supervision (equal); writing – review and editing (equal).

## FUNDING INFORMATION

This work was sponsored by the Natural Science Foundation of Shandong Province (ZR2022MH030).

## CONFLICT OF INTEREST STATEMENT

The authors declare no conflict of interest.

## INFORMED CONSENT STATEMENT

Informed consent was obtained from all subjects involved in the study.

## Supporting information


Appendix S1

## Data Availability

The data that support the findings of this study are available on request from the corresponding author.

## References

[fsn33801-bib-0001] Abrams, E. M. , & Sicherer, S. H. (2021). Cow's milk allergy prevention. Annals of Allergy, Asthma & Immunology, 127(1), 36–41. 10.1016/j.anai.2021.01.007 33450397

[fsn33801-bib-0002] Akagawa, S. , & Kaneko, K. (2022). Gut microbiota and allergic diseases in children. Allergology International, 71(3), 301–309. 35314107 10.1016/j.alit.2022.02.004

[fsn33801-bib-0003] Azad, M. B. , Konya, T. , Guttman, D. S. , Field, C. J. , Sears, M. R. , HayGlass, K. T. , Mandhane, P. J. , Turvey, S. E. , Subbarao, P. , Becker, A. B. , Scott, J. A. , Kozyrskyj, A. L. , & CHILD Study Investigators . (2015). Infant gut microbiota and food sensitization: Associations in the first year of life. Clinical and Experimental Allergy, 45(3), 632–643. 10.1111/cea.12487 25599982

[fsn33801-bib-0004] Berni Canani, R. , Nocerino, R. , Terrin, G. , Coruzzo, A. , Cosenza, L. , Leone, L. , & Troncone, R. (2012). Effect of *Lactobacillus GG* on tolerance acquisition in infants with cow's milk allergy: A randomized trial. The Journal of Allergy and Clinical Immunology, 129(2), 580–582, 582.e1‐5. 22078573 10.1016/j.jaci.2011.10.004

[fsn33801-bib-0005] Berni Canani, R. , Sangwan, N. , Stefka, A. T. , Nocerino, R. , Paparo, L. , Aitoro, R. , Calignano, A. , Khan, A. A. , Gilbert, J. A. , & Nagler, C. R. (2016). *Lactobacillus rhamnosus* GG‐supplemented formula expands butyrate‐producing bacterial strains in food allergic infants. The ISME Journal, 10(3), 742–750. 10.1038/ismej.2015.151 26394008 PMC4817673

[fsn33801-bib-0006] Burris, A. D. , Pizzarello, C. , & Järvinen, K. M. (2021). Immunologic components in human milk and allergic diseases with focus on food allergy. Seminars in Perinatology, 45(2), 151386. 33423794 10.1016/j.semperi.2020.151386

[fsn33801-bib-0007] Carr, L. E. , Virmani, M. D. , Rosa, F. , Munblit, D. , Matazel, K. S. , Elolimy, A. A. , & Yeruva, L. (2021). Role of human milk bioactives on infants' gut and immune health. Frontiers in Immunology, 12, 604080. 33643310 10.3389/fimmu.2021.604080 PMC7909314

[fsn33801-bib-0008] Chatchatee, P. , Nowak‐Wegrzyn, A. , Lange, L. , Benjaponpitak, S. , Chong, K. W. , Sangsupawanich, P. , van Ampting, M. T. J. , Oude Nijhuis, M. M. , Harthoorn, L. F. , Langford, J. E. , Knol, J. , Knipping, K. , Garssen, J. , Trendelenburg, V. , Pesek, R. , Davis, C. M. , Muraro, A. , Erlewyn‐Lajeunesse, M. , Fox, A. T. , Michaelis, L. J. , … PRESTO study team . (2022). Tolerance development in cow's milk‐allergic infants receiving amino acid‐based formula: A randomized controlled trial. The Journal of Allergy and Clinical Immunology, 149(2), 650–658.e5. 10.1016/j.jaci.2021.06.025 34224785

[fsn33801-bib-0009] Chen, C. , Liu, C. , Zhang, K. , & Xue, W. (2023). The role of gut microbiota and its metabolites short‐chain fatty acids in food allergy. Food Science and Human Wellness, 12(3), 702–710.

[fsn33801-bib-0010] Chen, C. C. , Chen, K. J. , Kong, M. S. , Chang, H. J. , & Huang, J. L. (2016). Alterations in the gut microbiotas of children with food sensitization in early life. Pediatric Allergy and Immunology, 27(3), 254–262. 26663491 10.1111/pai.12522

[fsn33801-bib-0011] D'Auria, E. , Mameli, C. , Piras, C. , Cococcioni, L. , Urbani, A. , Zuccotti, G. V. , & Roncada, P. (2018). Precision medicine in cow's milk allergy: Proteomics perspectives from allergens to patients. Journal of Proteomics, 188, 173–180. 29408543 10.1016/j.jprot.2018.01.018

[fsn33801-bib-0012] de Vos, W. M. , Tilg, H. , Van Hul, M. , & Cani, P. D. (2022). Gut microbiome and health: Mechanistic insights. Gut, 71(5), 1020–1032. 35105664 10.1136/gutjnl-2021-326789 PMC8995832

[fsn33801-bib-0013] Feehley, T. , Plunkett, C. H. , Bao, R. , Choi Hong, S. M. , Culleen, E. , Belda‐Ferre, P. , Campbell, E. , Aitoro, R. , Nocerino, R. , Paparo, L. , Andrade, J. , Antonopoulos, D. A. , Berni Canani, R. , & Nagler, C. R. (2019). Healthy infants harbor intestinal bacteria that protect against food allergy. Nature Medicine, 25(3), 448–453. 10.1038/s41591-018-0324-z PMC6408964 30643289

[fsn33801-bib-0014] Gamirova, A. , Berbenyuk, A. , Levina, D. , Peshko, D. , Simpson, M. R. , Azad, M. B. , Järvinen, K. M. , Brough, H. A. , Genuneit, J. , Greenhawt, M. , Verhasselt, V. , Peroni, D. G. , Perkin, M. R. , Warner, J. O. , Palmer, D. J. , Boyle, R. J. , & Munblit, D. (2022). Food proteins in human breast milk and probability of IgE‐mediated allergic reaction in children during breastfeeding: A systematic review. The Journal of Allergy and Clinical Immunology. In Practice, 10(5), 1312–1324.e8. 10.1016/j.jaip.2022.01.028 35123103

[fsn33801-bib-0015] Guest, J. F. , & Fuller, G. W. (2019). Effectiveness of using an extensively hydrolyzed casein formula supplemented with *Lactobacillus rhamnosus* GG compared with an extensively hydrolysed whey formula in managing cow's milk protein allergic infants. Journal of Comparative Effectiveness Research, 8(15), 1317–1326. 31526139 10.2217/cer-2019-0088

[fsn33801-bib-0016] Gupta, R. S. , Warren, C. M. , Smith, B. M. , Blumenstock, J. A. , Jiang, J. , Davis, M. M. , & Nadeau, K. C. (2018). The public health impact of parent‐reported childhood food allergies in the United States. Pediatrics, 142(6), e20181235. 30455345 10.1542/peds.2018-1235 PMC6317772

[fsn33801-bib-0017] Gupta, R. S. , Warren, C. M. , Smith, B. M. , Jiang, J. , Blumenstock, J. A. , Davis, M. M. , Schleimer, R. P. , & Nadeau, K. C. (2019). Prevalence and severity of food allergies among US adults. JAMA Network Open, 2(1), e185630. 10.1001/jamanetworkopen.2018.5630 30646188 PMC6324316

[fsn33801-bib-0018] Kok, C. R. , Brabec, B. , Chichlowski, M. , Harris, C. L. , Moore, N. , Wampler, J. L. , Vanderhoof, J. , Rose, D. , & Hutkins, R. (2020). Stool microbiome, pH and short/branched chain fatty acids in infants receiving extensively hydrolyzed formula, amino acid formula, or human milk through two months of age. BMC Microbiology, 20(1), 337. 10.1186/s12866-020-01991-5 33167908 PMC7650147

[fsn33801-bib-0019] Kordy, K. , Gaufin, T. , Mwangi, M. , Li, F. , Cerini, C. , Lee, D. J. , Adisetiyo, H. , Woodward, C. , Pannaraj, P. S. , Tobin, N. H. , & Aldrovandi, G. M. (2020). Contributions to human breast milk microbiome and enteromammary transfer of *Bifidobacterium breve* . PLoS One, 15(1), e0219633. 10.1371/journal.pone.0219633 31990909 PMC6986747

[fsn33801-bib-0020] Kostadinova, A. I. , Meulenbroek, L. A. , van Esch, B. C. , Hofman, G. A. , Garssen, J. , Willemsen, L. E. , & Knippels, L. M. (2016). A specific mixture of fructo‐oligosaccharides and *Bifidobacterium breve M‐16V* facilitates partial non‐responsiveness to whey protein in mice orally exposed to β‐lactoglobulin‐derived peptides. Frontiers in Immunology, 7, 673. 28127297 10.3389/fimmu.2016.00673 PMC5226939

[fsn33801-bib-0021] Lajnaf, R. , Feki, S. , Ben Ameur, S. , Attia, H. , Kammoun, T. , Ayadi, M. A. , & Masmoudi, H. (2023). Cow's milk alternatives for children with cow's milk protein allergy ‐ review of health benefits and risks of allergic reaction. International Dairy Journal, 141, 105624.

[fsn33801-bib-0022] Lopes, J. P. , & Sicherer, S. (2020). Food allergy: Epidemiology, pathogenesis, diagnosis, prevention, and treatment. Current Opinion in Immunology, 66, 57–64. 32446135 10.1016/j.coi.2020.03.014

[fsn33801-bib-0023] Lyons, K. E. , Ryan, C. A. , Dempsey, E. M. , Ross, R. P. , & Stanton, C. (2020). Breast milk, a source of beneficial microbes and associated benefits for infant health. Nutrients, 12(4), 1039. 10.3390/nu12041039 32283875 PMC7231147

[fsn33801-bib-0024] Marchesi, J. R. , Adams, D. H. , Fava, F. , Hermes, G. D. , Hirschfield, G. M. , Hold, G. , Quraishi, M. N. , Kinross, J. , Smidt, H. , Tuohy, K. M. , Thomas, L. V. , Zoetendal, E. G. , & Hart, A. (2016). The gut microbiota and host health: A new clinical frontier. Gut, 65(2), 330–339. 10.1136/gutjnl-2015-309990 26338727 PMC4752653

[fsn33801-bib-0025] Moossavi, S. , Sepehri, S. , Robertson, B. , Bode, L. , Goruk, S. , Field, C. J. , Lix, L. M. , de Souza, R. J. , Becker, A. B. , Mandhane, P. J. , Turvey, S. E. , Subbarao, P. , Moraes, T. J. , Lefebvre, D. L. , Sears, M. R. , Khafipour, E. , & Azad, M. B. (2019). Composition and variation of the human milk microbiota are influenced by maternal and early‐life factors. Cell Host & Microbe, 25(2), 324–335.e4. 10.1016/j.chom.2019.01.011 30763539

[fsn33801-bib-0026] Pham, V. T. , Lacroix, C. , Braegger, C. P. , & Chassard, C. (2017). Lactate‐utilizing community is associated with gut microbiota dysbiosis in colicky infants. Scientific Reports, 7(1), 11176. 28894218 10.1038/s41598-017-11509-1 PMC5593888

[fsn33801-bib-0027] Sagar, S. , Vos, A. P. , Morgan, M. E. , Garssen, J. , Georgiou, N. A. , Boon, L. , Kraneveld, A. D. , & Folkerts, G. (2014). The combination of *Bifidobacterium breve* with non‐digestible oligosaccharides suppresses airway inflammation in a murine model for chronic asthma. Biochimica et Biophysica Acta, 1842(4), 573–583. 10.1016/j.bbadis.2014.01.005 24440361

[fsn33801-bib-0028] Savage, J. H. , Lee‐Sarwar, K. A. , Sordillo, J. , Bunyavanich, S. , Zhou, Y. , O'Connor, G. , Sandel, M. , Bacharier, L. B. , Zeiger, R. , Sodergren, E. , Weinstock, G. M. , Gold, D. R. , Weiss, S. T. , & Litonjua, A. A. (2018). A prospective microbiome‐wide association study of food sensitization and food allergy in early childhood. Allergy, 73(1), 145–152. 10.1111/all.13232 28632934 PMC5921051

[fsn33801-bib-0029] Seth, D. , Poowutikul, P. , Pansare, M. , & Kamat, D. (2020). Food allergy: A review. Pediatric Annals, 49(1), e50–e58. 31930423 10.3928/19382359-20191206-01

[fsn33801-bib-0030] Shu, S. A. , Yuen, A. W. T. , Woo, E. , Chu, K. H. , Kwan, H. S. , Yang, G. X. , Yang, Y. , & Leung, P. S. C. (2019). Microbiota and food allergy. Clinical Reviews in Allergy and Immunology, 57(1), 83–97. 10.1007/s12016-018-8723-y 30564985

[fsn33801-bib-0031] Strisciuglio, C. , Vitale, A. , Perna, F. , Garziano, F. , Dolce, P. , Vitale, S. , Micillo, T. , Oglio, F. , Del Giudice, M. M. , Matarese, G. , & Gianfrani, C. (2023). Bifidobacteria modulate immune response in pediatric patients with cow's milk protein allergy. Pediatric Research, 94(3), 1111–1118. 10.1038/s41390-023-02534-0 36959319

[fsn33801-bib-0032] Tang, R. , Lyu, X. , Liu, Y. , Zhu, M. , Yang, X. , Wu, Z. , Han, B. , Wu, S. , & Sun, J. (2022). Four clinical phenotypes of cow's milk protein allergy based on dairy product specific IgE antibody types in North China. Frontiers in Immunology, 13, 949629. 10.3389/fimmu.2022.949629 36275773 PMC9585381

[fsn33801-bib-0033] Thompson‐Chagoyan, O. C. , Fallani, M. , Maldonado, J. , Vieites, J. M. , Khanna, S. , Edwards, C. , Doré, J. , & Gil, A. (2011). Faecal microbiota and short‐chain fatty acid levels in faeces from infants with cow's milk protein allergy. International Archives of Allergy and Immunology, 156(3), 325–332. 10.1159/000323893 21720179

[fsn33801-bib-0034] Triantis, V. , Bode, L. , & van Neerven, R. J. J. (2018). Immunological effects of human milk oligosaccharides. Frontiers in Pediatrics, 6, 190. 30013961 10.3389/fped.2018.00190 PMC6036705

[fsn33801-bib-0035] Verduci, E. , D'Elios, S. , Cerrato, L. , Comberiati, P. , Calvani, M. , Palazzo, S. , Martelli, A. , Landi, M. , Trikamjee, T. , & Peroni, D. G. (2019). Cow's milk substitutes for children: Nutritional aspects of milk from different mammalian species, special formula and plant‐based beverages. Nutrients, 11(8), 1739. 10.3390/nu11081739 31357608 PMC6723250

[fsn33801-bib-0036] Wang, G. , Yu, X. , Cong, Y. , & Li, L. (2022). Cow milk αs1‐casein induces allergic responses in a mouse model of atopy. Food Science and Human Wellness, 11(5), 1282–1289.

[fsn33801-bib-0037] Wang, Y. , Li, Z. , Wu, J. L. , Zhang, L. , Liu, M. , Tan, M. , Botma, A. , Liu, M. , Mulder, K. A. , Abrahamse‐Berkeveld, M. , & Cai, W. (2021). A partially hydrolyzed formula with synbiotics supports adequate growth and is well tolerated in healthy, Chinese term infants: A double‐blind, randomized controlled trial. Nutrition, 91‐92, 111472. 10.1016/j.nut.2021.111472 34626956

[fsn33801-bib-0038] Wopereis, H. , van Ampting, M. T. J. , Cetinyurek‐Yavuz, A. , Slump, R. , Candy, D. C. A. , Butt, A. M. , Peroni, D. G. , Vandenplas, Y. , Fox, A. T. , Shah, N. , Roeselers, G. , Harthoorn, L. F. , Michaelis, L. J. , Knol, J. , West, C. E. , & ASSIGN study group . (2019). A specific synbiotic‐containing amino acid‐based formula restores gut microbiota in non‐IgE mediated cow's milk allergic infants: A randomized controlled trial. Clin Transl Allergy, 9, 27. 10.1186/s13601-019-0267-6 31164972 PMC6543596

[fsn33801-bib-0039] Yamamoto‐Hanada, K. , Sato, M. , Toyokuni, K. , Irahara, M. , Hiraide‐Kotaki, E. , Harima‐Mizusawa, N. , Morita, H. , Matsumoto, K. , & Ohya, Y. (2023). Combination of heat‐killed *Lactiplantibacillus plantarum* YIT 0132 (LP0132) and oral immunotherapy in cow's milk allergy: A randomised controlled trial. Benef Microbes, 14(1), 17–29. 10.3920/BM2022.0064 36815492

[fsn33801-bib-0040] Yu, Z. , Yue, L. , Yang, Z. , Wang, Y. , Wang, Z. , Zhou, F. , Li, C. , Li, L. , Zhang, W. , & Li, X. (2023). Impairment of intestinal barrier associated with the alternation of intestinal flora and its metabolites in cow's milk protein allergy. Microbial Pathogenesis, 183, 106329. 10.1016/j.micpath.2023.106329 37659726

[fsn33801-bib-0042] Zhao, Y. , Bi, J. , Yi, J. , Peng, J. , & Ma, Q. (2022). Dose‐dependent effects of apple pectin on alleviating high fat‐induced obesity modulated by gut microbiota and SCFAs. Food Science and Human Wellness, 11(1), 143–154.

[fsn33801-bib-0043] Zhu, J. , & Dingess, K. A. (2019). The functional power of the human milk proteome. Nutrients, 11(8), 1834. 10.3390/nu11081834 31398857 PMC6723708

