# Supplementary material for: Alterations in the gut microbiota of toddlers with cow milk protein allergy treated with a partially hydrolyzed formula containing synbiotics: A nonrandomized controlled interventional study
Source: Food Sci Nutr. 2023 Nov 14;12(2):765–75. doi: 10.1002/fsn3.3801 (PMC10867501; doi:10.1002/fsn3.3801)

*Supporting information for*

**Alterations in the gut microbiota of toddlers with cow milk protein allergy treated with a partially hydrolyzed formula containing synbiotics: A nonrandomized controlled interventional study**

Mengyao Qian^1^, Wei Liu^1^, Xueying Feng^1^, Zhaochuan Yang^1^, Xiaomei Liu^1^, Liang Ma^1^, Yanchun Shan^1^, Ni Ran^1^, Mingji Yi^1^, Changlong Wei^3^, Chenyang Lu^2^, Yanxia Wang^1^*

^1^ The Affiliated Hospital of Qingdao University, Qingdao, China

^2^ School of Marine Science, Ningbo University, Ningbo, China

^3^ Shanghai Genecobio Company Ltd., Shanghai, China

*** Corresponding author**

Dr., Yanxia Wang

E-mail address: wangyx@qduhospital.cn

Postal address: The affiliated hospital of Qingdao University, 16 Jiangsu Road, Shinan District, Qingdao 266003, China

**Table S1.** Compositions of the partially hydrolyzed formula used in this study.

| **Composition** | **Content** | **Composition** | **Content** |
| --- | --- | --- | --- |
| **Energy** (Kcal/100 ml) | 64 | **Minerals** |  |
| **Fat** (g/100 ml) | 2.9 | Potassium (mg/100 ml) | 44 |
| Linoleic acid (mg/100 ml) | 310 | Iodine (mg/100 ml) | 7.6 |
| α-Linolenic acid (mg/100 ml) | 57 | Zinc (mg/100 ml) | 0.3 |
| DHA (mg/100 ml) | 5.4 | **Vitamins** |  |
| ARA (mg/100 ml) | 5.4 | Vitamin A (μgRE/100 ml) | 50 |
| **Protein** (g/100ml) | 1.5 | Vitamin D (μg/100 ml) | 1.1 |
| Whey | 1.5 | Vitamin E (mg α-TE/100 ml) | 0.51 |
| **Carbohydrates** (g/100ml) | 8.0 | Vitamin K (μg/100 ml) | 1.8 |
| scGOS and lcFOS (g/100ml) | 0.8 | Vitamin B_1_ (μg/100 ml) | 25 |
| ***Bifidobacterium breve* M-16V** | √ | Vitamin B_2_ (μg/100 ml) | 57 |
| **Minerals** |  | Vitamin B_3_ (μg/100 ml) | 213 |
| Iron (mg/100 ml) | 0.61 | Vitamin B_5_ (μg/100 ml) | 166 |
| Calcium (mg/100 ml) | 42 | Vitamin B_6_ (μg/100 ml) | 20 |
| Phosphorus (mg/100 ml) | 23 | Vitamin B_12_ (μg/100 ml) | 0.033 |
| Magnesium (mg/100 ml) | 3.0 | Vitamin C (mg/100 ml) | 4.5 |
| Sodium (mg/100 ml) | 29 | Folic acid (μg/100 ml) | 4.1 |
| Chlorine (mg/100 ml) | 25 | Biotin (μg/100 ml) | 0.84 |

scGOS, short-chain galacto-oligosaccharides; lcFOS, long-chain fructooligosaccharides.

**Table S2**. Bacterial taxonomic profiling at the phylum level in allergic toddlers fed with pHF for 3 months. Data was represented as mean ± SD.

|  | **pHF-0m** | **pHF-1m** | **pHF-3m** |
| --- | --- | --- | --- |
| Firmicutes | 0.442±0.203 | 0.455±0.234 | 0.468±0.185 |
| Actinobacteriota | 0.372±0.259 | 0.328±0.253 | 0.318±0.206 |
| Bacteroidota | 0.166±0.155 | 0.181±0.172 | 0.19±0.161 |
| Proteobacteria | 0.019±0.015 | 0.033±0.054 | 0.023±0.021 |
| others | 0.002±0.004 | 0.003±0.01 | 0.001±0.002 |

**Table S3**. Bacterial taxonomic profiling at the class level in allergic toddlers fed with pHF for 3 months. Data was represented as mean ± SD. Different lowercase letters represent signiﬁcant diﬀerences by one-way ANOVA (*p*<0.05).

|  | **pHF-0m** | **pHF-1m** | **pHF-3m** |
| --- | --- | --- | --- |
| Clostridia | 0.361±0.227 | 0.369±0.214 | 0.428±0.175 |
| Actinobacteria | 0.321±0.252 | 0.354±0.256 | 0.295±0.2 |
| Bacteroidia | 0.181±0.171 | 0.166±0.155 | 0.19±0.161 |
| Bacilli | 0.065±0.174^ab^ | 0.027±0.032^a^ | 0.016±0.015^b^ |
| Negativicutes | 0.03±0.042 | 0.046±0.078 | 0.024±0.056 |
| Gammaproteobacteria | 0.033±0.054 | 0.018±0.015 | 0.023±0.021 |
| Coriobacteriia | 0.007±0.017 | 0.018±0.051 | 0.023±0.055 |
| others | 0.003±0.01 | 0.002±0.004 | 0.001±0.002 |

**Table S4**. STEM analysis of 74 key OTUs with high abundance identified in allergic toddlers fed with pHF for 0, 1 and 3 months. Data was represented as mean. OTUs grouped into different clusters are marked with different colors, as shown in Fig. 2C.

| **OTU ID** | **pHF-0m** | **pHF-1m** | **pHF-3m** | **Genus** |
| --- | --- | --- | --- | --- |
| OTU867 | 0.173784394 | 0.158072516 | 0.111775693 | *Bifidobacterium* |
| OTU902 | 0.080377712 | 0.129193119 | 0.134421462 | *Bifidobacterium* |
| OTU917 | 0.071136821 | 0.090588446 | 0.110470755 | *Faecalibacterium* |
| OTU657 | 0.062150473 | 0.052637854 | 0.05368073 | *Bacteroides* |
| OTU970 | 0.054331587 | 0.047423473 | 0.041150106 | *Bifidobacterium* |
| OTU850 | 0.032870465 | 0.027816114 | 0.026489696 | *Bacteroides* |
| OTU852 | 0.02402911 | 0.027258697 | 0.017137107 | *Eubacterium_eligens_group* |
| OTU864 | 0.016307961 | 0.021106694 | 0.026576692 | *Fusicatenibacter* |
| **OTU1151** | 0.01663339 | 0.014702297 | 0.0311284 | *Agathobacter* |
| OTU884 | 0.013952629 | 0.016878267 | 0.023286961 | *Blautia* |
| OTU888 | 0.023210705 | 0.013316808 | 0.016530284 | *Ruminococcus_gnavus_group* |
| **OTU879** | 0.005797575 | 0.017216584 | 0.022514738 | *Collinsella* |
| OTU907 | 0.01419321 | 0.014449902 | 0.012181566 | *Lachnospira* |
| **OTU719** | 0.00274198 | 0.002929934 | 0.032698622 | *Bacteroides* |
| **OTU912** | 0.003418614 | 0.026239449 | 0.008035838 | *Prevotella* |
| **OTU869** | 0.015260789 | 0.013295327 | 0.005882422 | *Veillonella* |
| OTU1154 | 0.009740312 | 0.012474774 | 0.01095718 | *Ruminococcus_torques_group* |
| **OTU302** | 0.014027811 | 0.010232214 | 0.0065118 | *Bacteroides* |
| **OTU1153** | 0.010808965 | 0.015860094 | 0.003806337 | *Bifidobacterium* |
| **OTU889** | 0.018255164 | 0.006399027 | 0.004910432 | *Escherichia-Shigella* |
| **OTU301** | 0.014597043 | 0.004766513 | 0.009490065 | *Bacteroides* |
| OTU709 | 0.006546168 | 0.009815494 | 0.011554336 | *Lachnospira* |
| **OTU880** | 0.016785901 | 0.005568808 | 0.004370198 | *Erysipelatoclostridium* |
| OTU887 | 0.010517904 | 0.009670501 | 0.006013453 | *Clostridium_sensu_stricto_1* |
| **OTU650** | 0.024994657 | 0.000629377 | 0.000457534 | *Enterococcus* |
| OTU556 | 0.006800712 | 0.007527825 | 0.011374975 | *Lachnospiraceae_UCG-004* |
| OTU915 | 0.00776948 | 0.007064921 | 0.008069133 | *Anaerostipes* |
| **OTU230** | 0.004378791 | 0.011836804 | 0.006281959 | *Alistipes* |
| **OTU1277** | 0.006328142 | 0.004596817 | 0.00927526 | *Bacteroides* |
| **OTU1279** | 0.002948192 | 0.007438681 | 0.009776829 | *Parasutterella* |
| OTU580 | 0.005206862 | 0.00716588 | 0.006899522 | *Lachnoclostridium* |
| **OTU1403** | 0.000878551 | 0.010598456 | 0.007601933 | *Megamonas* |
| **OTU1203** | 0.001933241 | 0.011920578 | 0.004851361 | *Megasphaera* |
| OTU862 | 0.004370198 | 0.007779147 | 0.006353918 | *Lachnospiraceae_ND3007_group* |
| **OTU1385** | 0.011213871 | 0.00113739 | 0.005132755 | *Prevotella* |
| OTU885 | 0.007010146 | 0.003878296 | 0.00358294 | *Streptococcus* |
| **OTU871** | 0.002777423 | 0.002008422 | 0.009615725 | *Romboutsia* |
| **OTU848** | 0.008573923 | 0.003156553 | 0.00237896 | *Lachnoclostridium* |
| OTU896 | 0.003531387 | 0.005476442 | 0.00470959 | *Roseburia* |
| OTU916 | 0.005672988 | 0.004003957 | 0.003834261 | *unclassified_f__Lachnospiraceae* |
| **OTU969** | 0.006281959 | 0.002957859 | 0.003252141 | *Hungatella* |
| **OTU906** | 0.006480653 | 0.001765693 | 0.004226279 | *Haemophilus* |
| OTU567 | 0.003097482 | 0.003624827 | 0.005325005 | *Roseburia* |
| **OTU1188** | 0.002078234 | 0.004602187 | 0.00503287 | *CAG-352* |
| OTU1157 | 0.002876233 | 0.005157457 | 0.003454057 | *Erysipelotrichaceae_UCG-003* |
| **OTU1136** | 0.005545179 | 0.002738758 | 0.002871937 | *Lachnospiraceae_NK4A136_group* |
| OTU909 | 0.004476527 | 0.002728018 | 0.003536757 | *Eubacterium_hallii_group* |
| OTU656 | 0.002722648 | 0.002704389 | 0.005216528 | *Bacteroides* |
| OTU1285 | 0.004194059 | 0.002812866 | 0.003616234 | *Parabacteroides* |
| **OTU1135** | 0.002231819 | 0.004558152 | 0.003237104 | *Subdoligranulum* |
| **OTU914** | 0.005804019 | 0.001955795 | 0.00141234 | *Ruminococcus* |
| OTU577 | 0.002304853 | 0.002395071 | 0.00425313 | *Dorea* |
| **OTU595** | 0.0014016 | 0.003306916 | 0.003460501 | *Eubacterium_siraeum_group* |
| **OTU1256** | 0.003083519 | 0.001589554 | 0.00332947 | *Alistipes* |
| OTU910 | 0.002136231 | 0.002658206 | 0.002792459 | *Monoglobus* |
| **OTU911** | 0.00536904 | 0.001271643 | 0.000743224 | *Clostridium_innocuum_group* |
| OTU875 | 0.001825839 | 0.002189932 | 0.003276843 | *Ruminococcus_torques_group* |
| OTU1137 | 0.002221079 | 0.002149119 | 0.002914898 | *unclassified_f__Lachnospiraceae* |
| **OTU960** | 0.003886888 | 0.000998841 | 0.002382182 | *Blautia* |
| OTU849 | 0.003076001 | 0.002334925 | 0.001678698 | *Intestinibacter* |
| **OTU829** | 0.000759334 | 0.003622679 | 0.002548656 | *Bacteroides* |
| **OTU1067** | 0.000958028 | 0.002690427 | 0.003208106 | *Bifidobacterium* |
| OTU877 | 0.002420847 | 0.002133009 | 0.001947203 | *Flavonifractor* |
| **OTU596** | 0.00105469 | 0.003431503 | 0.00181295 | *unclassified_f__Lachnospiraceae* |
| OTU1229 | 0.003256437 | 0.003013708 | 0 | *Veillonella* |
| **OTU583** | 0.003231734 | 0.000361946 | 0.002424069 | *Lachnospiraceae_UCG-003* |
| OTU733 | 0.00130816 | 0.002015941 | 0.002540064 | *Bacteroides* |
| **OTU856** | 0.00281931 | 0.00196224 | 0.000791555 | *Clostridium_sensu_stricto_1* |
| **OTU1292** | 0.002146971 | 0.002304853 | 0.000971991 | *Megasphaera* |
| OTU870 | 0.001696956 | 0.001369379 | 0.00219208 | *Clostridium_sensu_stricto_1* |
| **OTU336** | 0.004963059 | 4.29609E-06 | 7.51816E-06 | *Prevotella* |
| **OTU197** | 0.000990249 | 0.002652836 | 0.00125231 | *Prevotella* |
| **OTU557** | 0.001451005 | 0.000824849 | 0.002564766 | *Lachnospiraceae_NK4A136_group* |
| **OTU1387** | 0.001053616 | 0.00125231 | 0.002479918 | *Blautia* |

**Table S5**. Bacterial taxonomic profiling at the phylum level in allergic and healthy toddlers fed with pHF, IF and HM at baseline and after 3 months. Data was represented as mean ± SD. Different lowercase letters represent signiﬁcant diﬀerences between 0m and 3m groups under same feeding by one-way ANOVA (*p*<0.05).

|  | **pHF** | | **IF** | | **HM** | |
| --- | --- | --- | --- | --- | --- | --- |
|  | **0m** | **3m** | **0m** | **3m** | **0m** | **3m** |
| Firmicutes | 0.442±0.203 | 0.468±0.185 | 0.372±0.184 | 0.429±0.17 | 0.393±0.179^a^ | 0.516±0.175^b^ |
| Actinobacteriota | 0.372±0.259 | 0.318±0.206 | 0.331±0.215 | 0.3±0.178 | 0.394±0.223 | 0.271±0.201 |
| Bacteroidota | 0.166±0.155 | 0.19±0.161 | 0.221±0.169 | 0.242±0.17 | 0.169±0.209 | 0.166±0.172 |
| Proteobacteria | 0.019±0.015 | 0.023±0.021 | 0.074±0.17 | 0.026±0.024 | 0.043±0.051 | 0.046±0.054 |
| others | 0.002±0.004 | 0.001±0.002 | 0.002±0.008 | 0.003±0.006 | 0.002±0.002 | 0.001±0.001 |

**Table S6**. Bacterial taxonomic profiling at the class level in allergic and healthy toddlers fed with pHF, IF and HM at baseline and after 3 months. Data was represented as mean ± SD. Different lowercase letters represent signiﬁcant diﬀerences between 0m and 3m groups under same feeding by one-way ANOVA (*p*<0.05).

|  | **pHF** | | **IF** | | **HM** | |
| --- | --- | --- | --- | --- | --- | --- |
|  | **0m** | **3m** | **0m** | **3m** | **0m** | **3m** |
| Clostridia | 0.361±0.227 | 0.428±0.175 | 0.329±0.183 | 0.386±0.16 | 0.293±0.141^a^ | 0.455±0.201^b^ |
| Actinobacteria | 0.321±0.252 | 0.295±0.2 | 0.325±0.215 | 0.284±0.177 | 0.376±0.224 | 0.26±0.2 |
| Bacteroidia | 0.181±0.171 | 0.19±0.161 | 0.221±0.169 | 0.242±0.17 | 0.169±0.209 | 0.166±0.172 |
| Bacilli | 0.065±0.174 | 0.016±0.015 | 0.028±0.03 | 0.026±0.026 | 0.047±0.092 | 0.022±0.015 |
| Negativicutes | 0.03±0.042 | 0.024±0.056 | 0.014±0.021 | 0.017±0.025 | 0.053±0.046 | 0.039±0.057 |
| Gammaproteobacteria | 0.033±0.054 | 0.023±0.021 | 0.074±0.17 | 0.026±0.024 | 0.043±0.051 | 0.046±0.054 |
| Coriobacteriia | 0.007±0.017 | 0.023±0.055 | 0.006±0.013^a^ | 0.016±0.031^b^ | 0.017±0.041 | 0.01±0.023 |
| others | 0.003±0.01 | 0.001±0.002 | 0.002±0.008 | 0.003±0.007 | 0.002±0.002 | 0.001±0.001 |

**Table S7**. Forty-eight genera with different abundances (fold change>4) in response to pHF feeding in allergic toddlers between baseline and 3 months were identified in this study. Data was represented as mean.

| **Genus** | **0m (%)** | **3m (%)** | **Fold Change (0m/3m)** |
| --- | --- | --- | --- |
| *Butyrivibrio* | 0.001 | 0.036 | 0.015 |
| *unclassified_o__Bacteroidales* | 0 | 0.007 | 0.031 |
| *Fournierella* | 0 | 0.009 | 0.037 |
| *Eubacterium* | 0 | 0.002 | 0.056 |
| *norank_f__norank_o__Clostridia_UCG-014* | 0.017 | 0.204 | 0.083 |
| *Allisonella* | 0.001 | 0.015 | 0.085 |
| *Acetitomaculum* | 0.002 | 0.014 | 0.107 |
| *Megamonas* | 0.096 | 0.767 | 0.122 |
| *Hafnia-Obesumbacterium* | 0 | 0.001 | 0.125 |
| *Leuconostoc* | 0 | 0.001 | 0.125 |
| *Howardella* | 0 | 0.001 | 0.143 |
| *Enterobacter* | 0.014 | 0.091 | 0.149 |
| *Odoribacter* | 0.001 | 0.004 | 0.154 |
| *Olsenella* | 0 | 0.002 | 0.167 |
| *Eubacterium_ruminantium_group* | 0.01 | 0.055 | 0.184 |
| *Butyricimonas* | 0 | 0.002 | 0.2 |
| *Leptotrichia* | 0 | 0.001 | 0.2 |
| *Family_XIII_UCG-001* | 0 | 0.001 | 0.2 |
| *UCG-003* | 0.011 | 0.043 | 0.246 |
| *Paludicola* | 0 | 0 | 4 |
| *Sphingomonas* | 0 | 0 | 4 |
| *norank_f__Eubacterium_coprostanoligenes_group* | 0.413 | 0.1 | 4.031 |
| *norank_f__Butyricicoccaceae* | 0.004 | 0.001 | 4.125 |
| *Rothia* | 0.027 | 0.006 | 4.698 |
| *Christensenellaceae_R-7_group* | 0.201 | 0.037 | 5.351 |
| *unclassified_p__Firmicutes* | 0.004 | 0.001 | 5.667 |
| *Paraprevotella* | 0.107 | 0.018 | 5.69 |
| *Helicobacter* | 0.001 | 0 | 6 |
| *Rhodococcus* | 0.008^a^ | 0.001^b^ | 6.545 |
| *Clostridium_innocuum_group* | 0.55 | 0.074 | 7.224 |
| *Robinsoniella* | 0.003 | 0 | 7.75 |
| *Burkholderia-Caballeronia-Paraburkholderia* | 0.022 | 0.003 | 7.769 |
| *Fusobacterium* | 0.193 | 0.024 | 7.848 |
| *Coprobacillus* | 0.011^a^ | 0.001^b^ | 8.909 |
| *Pseudoramibacter* | 0.001 | 0 | 9 |
| *Desulfovibrio* | 0.005 | 0.001 | 9.8 |
| *Peptoniphilus* | 0.002 | 0 | 10 |
| *norank_f__Muribaculaceae* | 0.021 | 0.002 | 10.105 |
| *Brevundimonas* | 0.002^a^ | 0^b^ | 14 |
| *Herbinix* | 0.006 | 0 | 14.25 |
| *Eubacterium_xylanophilum_group* | 0.03^a^ | 0.002^b^ | 15.333 |
| *Fenollaria* | 0.002 | 0 | 19 |
| *Actinobacillus* | 0.049 | 0.001 | 37.333 |
| *Enterococcus* | 2.562 | 0.046 | 54.47 |
| *Acidaminococcus* | 0.143 | 0.002 | 94 |
| *Anaerosporobacter* | 0.131 | 0.001 | 170.571 |
| *Epulopiscium* | 0.032^a^ | 0^b^ | 296 |
| *Anaeroglobus* | 0.042 | 0 | 381 |

**Table S8**. Thirty-eight genera with different abundances (fold change>4) in response to IF feeding in healthy toddlers between baseline and 3 months were identified in this study. Data was represented as mean.

| **Genus** | **0m (%)** | **3m (%)** | **Fold Change (0m/3m)** |
| --- | --- | --- | --- |
| *Anaerofustis* | 0 | 0.006 | 0.018 |
| *Fournierella* | 0.001 | 0.027 | 0.019 |
| *norank_f__Butyricicoccaceae* | 0 | 0.008 | 0.027 |
| *Phocea* | 0^a^ | 0.003^b^ | 0.033 |
| *Hafnia-Obesumbacterium* | 0 | 0.005 | 0.043 |
| *norank_f__Peptococcaceae* | 0 | 0.002 | 0.053 |
| *unclassified_f__Selenomonadaceae* | 0 | 0.004 | 0.053 |
| *Moryella* | 0 | 0.001 | 0.071 |
| *Lactobacillus* | 0.003 | 0.029 | 0.106 |
| *Weissella* | 0.001 | 0.008 | 0.113 |
| *Lachnospiraceae_UCG-010* | 0.003^a^ | 0.027^b^ | 0.127 |
| *Acetitomaculum* | 0 | 0.003 | 0.16 |
| *Peptoniphilus* | 0 | 0.001 | 0.167 |
| *Shuttleworthia* | 0 | 0.001 | 0.167 |
| *Delftia* | 0 | 0.001 | 0.167 |
| *norank_f__norank_o__RF39* | 0.003 | 0.018 | 0.169 |
| *Tyzzerella* | 0.014 | 0.081 | 0.17 |
| *CAG-56* | 0.004 | 0.02 | 0.203 |
| *Paraprevotella* | 0.002 | 0.01 | 0.214 |
| *Pseudoramibacter* | 0 | 0.001 | 0.222 |
| *unclassified_o__Oscillospirales* | 0.011 | 0.002 | 4.773 |
| *unclassified_f__Anaerovoracaceae* | 0.001 | 0 | 5 |
| *Terrisporobacter* | 0.01 | 0.002 | 5.706 |
| *Epulopiscium* | 0.015 | 0.003 | 5.769 |
| *Alloprevotella* | 0.005 | 0.001 | 6.714 |
| *Klebsiella* | 1.33 | 0.193 | 6.882 |
| *CHKCI001* | 0.009 | 0.001 | 6.917 |
| *Kluyvera* | 0.001 | 0 | 7 |
| *Escherichia-Shigella* | 4.239^a^ | 0.489^b^ | 8.677 |
| *Lachnoanaerobaculum* | 0.001 | 0 | 9 |
| *Acidaminococcus* | 0.021 | 0.002 | 9.182 |
| *norank_f__Christensenellaceae* | 0.002 | 0 | 11.5 |
| *Morganella* | 0.008 | 0.001 | 15 |
| *Anaerosporobacter* | 0.01 | 0.001 | 17 |
| *Howardella* | 0.002 | 0 | 23 |
| *Robinsoniella* | 0.02 | 0.001 | 28 |
| *unclassified_f__Enterobacteriaceae* | 0.147 | 0.002 | 65.273 |
| *Clostridium_sensu_stricto_2* | 0.206 | 0.001 | 200.7 |

**Table S9**. Forty-five genera with different abundances (fold change>4) in response to HM feeding in healthy toddlers between baseline and 3 months were identified in this study. Data was represented as mean.

| **Genus** | **0m (%)** | **3m (%)** | **Fold Change (0m/3m)** |
| --- | --- | --- | --- |
| *Lachnospiraceae_UCG-003* | 0.001 | 0.104 | 0.011 |
| *Aeromonas* | 0.001 | 0.051 | 0.022 |
| *Robinsoniella* | 0 | 0.006 | 0.037 |
| *Coprococcus* | 0.005 | 0.13 | 0.042 |
| *Phocaeicola* | 0.001 | 0.012 | 0.055 |
| *Weissella* | 0 | 0.008 | 0.057 |
| *Klebsiella* | 0.02 | 0.315 | 0.065 |
| *Anaerostignum* | 0 | 0.005 | 0.08 |
| *Eubacterium_nodatum_group* | 0 | 0.005 | 0.083 |
| *CAG-56* | 0.003 | 0.032 | 0.103 |
| *Anaerotruncus* | 0.002 | 0.016 | 0.108 |
| *Novosphingobium* | 0 | 0.002 | 0.111 |
| *unclassified_o__Oscillospirales* | 0.001 | 0.007 | 0.118 |
| *Ruminococcus_gauvreauii_group* | 0.031 | 0.211 | 0.149 |
| *Sellimonas* | 0.024 | 0.155 | 0.155 |
| *NK4A214_group* | 0.001 | 0.007 | 0.167 |
| *Lachnospiraceae_NC2004_group* | 0.01^a^ | 0.057^b^ | 0.173 |
| *Eubacterium_fissicatena_group* | 0.002 | 0.014 | 0.175 |
| *norank_f__Saccharimonadaceae* | 0 | 0.001 | 0.2 |
| *Providencia* | 0.002 | 0.011 | 0.2 |
| *Actinobacillus* | 0.002 | 0.011 | 0.212 |
| *unclassified_f__Ruminococcaceae* | 0.002 | 0.007 | 0.212 |
| *Ruminococcus* | 0.219 | 0.975 | 0.225 |
| *Dysgonomonas* | 0.014 | 0.062 | 0.23 |
| *Colidextribacter* | 0.035 | 0.149 | 0.232 |
| *Dorea* | 0.093 | 0.393 | 0.235 |
| *Lachnospiraceae_UCG-010* | 0.007 | 0.031 | 0.243 |
| *norank_f__Eubacterium_coprostanoligenes_group* | 0.104 | 0.421 | 0.247 |
| *Porphyromonas* | 0 | 0.002 | 0.25 |
| *norank_f__Oscillospiraceae* | 0.001 | 0.004 | 0.25 |
| *unclassified_f__Enterobacteriaceae* | 0.015 | 0.003 | 4.714 |
| *Akkermansia* | 0.064 | 0.014 | 4.726 |
| *Negativibacillus* | 0.001 | 0 | 5 |
| *Lactobacillus* | 0.34 | 0.067 | 5.109 |
| *Aggregatibacter* | 0.011^a^ | 0.002^b^ | 5.556 |
| *Olsenella* | 0.003 | 0 | 6 |
| *Enterococcus* | 0.517 | 0.08 | 6.441 |
| *Anaerococcus* | 0.002 | 0 | 7 |
| *Megasphaera* | 1.175 | 0.134 | 8.741 |
| *Hafnia-Obesumbacterium* | 0.004 | 0 | 16 |
| *Lactococcus* | 0.012 | 0.001 | 18 |
| *Lachnospiraceae_FCS020_group* | 0.063 | 0.002 | 36 |
| *Epulopiscium* | 0.111 | 0.002 | 50.4 |
| *Terrisporobacter* | 0.053 | 0.001 | 60 |
| *Kluyvera* | 0.049 | 0 | 225 |

**Table S10**. Abundance of 11 probiotics and SCFA producers in allergic and healthy toddlers fed with pHF, IF and HM at different time points. Data was represented as mean ± SD. Different lowercase letters represent signiﬁcant diﬀerences by one-way ANOVA (*p*<0.05).

|  | **pHF (%)** | | | **IF (%)** | | | **HM (%)** | | |
| --- | --- | --- | --- | --- | --- | --- | --- | --- | --- |
|  | **0m** | **1m** | **3m** | **0m** | **1m** | **3m** | **0m** | **1m** | **3m** |
| ***Bifidobacterium*** | 29.45±20.04 | 32.1±25.24 | 35.38±25.61 | 32.46±21.55 | 29.99±17.81 | 28.36±17.71 | 37.56±22.35 | 32.12±21.46 | 26.01±19.96 |
| ***Enterococcus*** | 0.04±0.12 | 2.5±14.93 | 0.06±0.16 | 0.08±0.22 | 0.05±0.1 | 0.03±0.07 | 0.54±1.86 | 0.11±0.22 | 0.08±0.24 |
| ***Lactobacillus*** | 0.16±0.74 | 0.42±0.84 | 0.74±2.16 | 0±0.01 | 0.01±0.02 | 0.03±0.19 | 0.35±0.68 | 0.3±0.65 | 0.07±0.14 |
| ***Streptococcus*** | 0.43±0.52 | 0.86±1.71 | 0.46±1.38 | 0.69±1.54 | 0.9±2.54 | 0.7±1.18 | 0.95±0.93 | 1.18±1.68 | 0.63±0.52 |
| ***Anaerostipes*** | 0.81±1.26 | 0.8±1.73 | 0.73±1.19 | 0.52±0.68 | 0.51±0.57 | 0.62±0.65 | 0.37±0.73 | 0.32±0.42 | 0.49±0.62 |
| ***Butyricicoccus*** | 0.1±0.2 | 0.09±0.14 | 0.08±0.1 | 0.08±0.13 | 0.17±0.42 | 0.11±0.14 | 0.21±0.32 | 0.23±0.51 | 0.2±0.26 |
| ***Faecalibacterium*** | 11.28±8.2^a^ | 7.22±9.46^b^ | 9.14±8.76^ab^ | 7.43±6.98 | 7.33±5.98 | 9.07±7.29 | 4.99±5.57^a^ | 8.34±6.95^ab^ | 10.03±8.35^b^ |
| ***Megasphaera*** | 0.6±2.86 | 0.41±1.36 | 1.44±4.2 | 0.09±0.37 | 0.03±0.11 | 0.13±0.59 | 1.18±2.96 | 1±2.61 | 0.14±0.21 |
| ***Roseburia*** | 1.17±1.47 | 0.72±1.53 | 1.09±2.06 | 1.53±2.74 | 1.3±1.78 | 1.26±1.91 | 1.48±3.15 | 1.05±3.01 | 3.5±6.37 |
| ***Ruminococcus*** | 0.27±0.42 | 0.77±2.41 | 0.5±1.28 | 0.31±0.54 | 0.64±1.36 | 0.36±0.62 | 0.22±0.44 | 0.27±0.54 | 0.99±1.88 |
| ***Subdoligranulum*** | 0.37±0.76 | 0.33±0.86 | 0.57±1.34 | 0.61±1.32 | 0.46±0.89 | 0.56±1.12 | 0.4±0.83 | 0.69±1.27 | 0.48±0.75 |

* The genera belonging to SCFA producers are marked with green, the genera belonging to probiotics are marked with red, and the genera regarded as both SCFA producers and probiotics are marked with blue.

**Fig S1.** Key genera with different abundances (fold change>4) in response to pHF, IF and breast milk feeding between baseline and 3 months of feeding. Key genera identified in the pHF (**A**), IF (**B**) and HM (**C**) groups. **D.** Venn diagram results of key genera identified in different pairwise comparisons. The heatmap shows the relative abundances of genera.


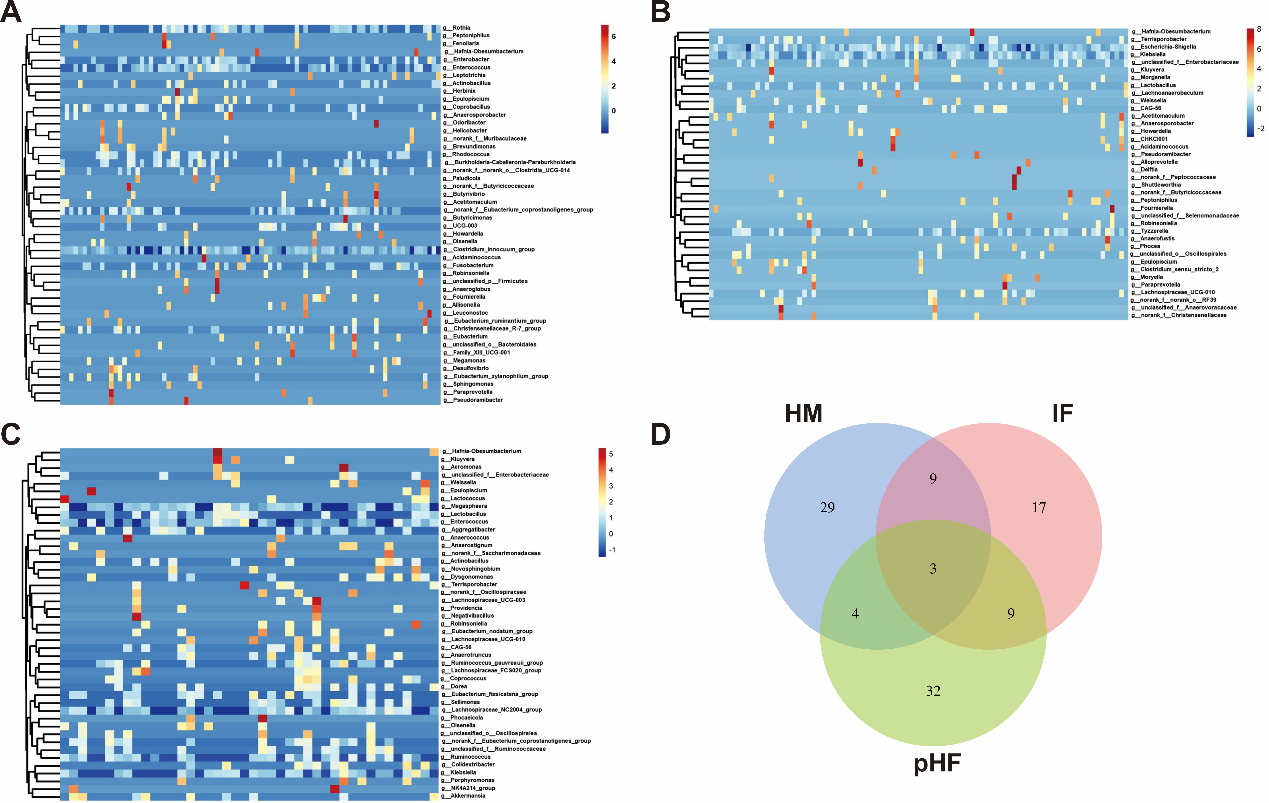

Supplement: Supplementary file 1 — Appendix S1 [file FSN3-12-765-s001.docx]
